# Influence of Food on Paediatric Gastrointestinal Drug Absorption Following Oral Administration: A Review

**DOI:** 10.3390/children2020244

**Published:** 2015-06-09

**Authors:** Hannah K. Batchelor

**Affiliations:** Pharmacy and Therapeutics, School of Clinical and Experimental Medicine, College of Medical and Dental Sciences, Medical School Building, University of Birmingham, Edgbaston B15 2TT, UK; E-Mail: h.k.batchelor@bham.ac.uk; Tel.: +44-121-414-3717

**Keywords:** paediatric, food–drug interaction, food effect

## Abstract

The objective of this paper was to review existing information regarding food effects on drug absorption within paediatric populations. Mechanisms that underpin food–drug interactions were examined to consider potential differences between adult and paediatric populations, to provide insights into how this may alter the pharmacokinetic profile in a child. Relevant literature was searched to retrieve information on food–drug interaction studies undertaken on: (i) paediatric oral drug formulations; and (ii) within paediatric populations. The applicability of existing methodology to predict food effects in adult populations was evaluated with respect to paediatric populations where clinical data was available. Several differences in physiology, anatomy and the composition of food consumed within a paediatric population are likely to lead to food–drug interactions that cannot be predicted based on adult studies. Existing methods to predict food effects cannot be directly extrapolated to allow predictions within paediatric populations. Development of systematic methods and guidelines is needed to address the general lack of information on examining food–drug interactions within paediatric populations.

## 1. Introduction

Clinical studies that measure food–drug interactions are critical to evaluate appropriate dosing, timing, and formulation of new drug candidates. There are several excellent reviews on food effects in adult populations (e.g., [1]).

In paediatric populations there is evidence that a wide range of drugs are mixed with food prior to administration to ensure that medication is acceptable to the patient [2]. The British National Formulary for Children (BNF-C) [3] lists 11 drugs that are recommended to be mixed with food to assist with administration; specific foods mentioned include soft foods, yoghurt, apple sauce, jam or honey. The BNF-C also lists at least eight examples of medicines suggested to be mixed with fruit juice prior to administration [3].

The extrapolation of fed effects observed in adults into paediatric populations is an unexplored and complex area. There are key differences between the feeding patterns of paediatric patients and adults both in terms of food composition and feeding frequency. In addition, the gastrointestinal tract of paediatric patients can be different to that found in adults. This review seeks to highlight potential differences in fed effects in paediatric patients compared to adults by reviewing existing data sources on physiological/anatomical, physicochemical and formulation differences.

## 2. Clinical Measurement of Food–Drug Interactions

Clinically significant food–drug interactions are usually assessed in terms of peak plasma concentration (Cmax), time to Cmax (Tmax) and area under the absorption time curve (AUC) in a plot of plasma concentration against time. Differences in the rate of absorption (faster or slower) will alter Tmax yet are unlikely to affect Cmax or AUC. Differences in the extent of absorption will affect Cmax and AUC which typically have greater clinical significance compared to changes in Tmax.

### 2.1. Regulatory Guidelines for Food–Drug Interaction Studies

Food–drug interactions are widely reported in adult populations with regulatory bodies recognising their significance with dedicated guidance on the conduct of fed effect clinical studies [4]. In terms of regulatory guidance, a clinically significant food effect is defined as one where the 90 percent confidence interval fails to meet the limits of 80–125 percent for either Cmax or AUC of the fasted profile [4].

For paediatric populations the guidance surrounding food effects is limited. Based on US law, the “pediatric study decision tree” [5] allows extrapolation from adult data sets if there is sufficient similarity of both: (i) disease progression; and (ii) response to intervention between source and target population. If the exposure-response relationship of the medicinal product is assumed to be similar, the only PK studies required in paediatric populations, are those for dose determination and safety evaluation. This logic is also replicated in European Medicines Agency (EMA) guidance where it is stated that relative bioavailability comparisons of paediatric formulations with the adult oral formulation should typically be conducted in adults; with only dose selection pharmacokinetic studies required in paediatric populations [6]. This results in the majority of paediatric pharmacokinetic studies being conducted in the fasted state with very limited pharmacokinetic studies in the fed state. Indeed neither the EMA guideline on the role of pharmacokinetics in the development of medicinal products in the paediatric population nor the International Conference on Harmonisation (ICH) topic on clinical investigation of medicinal products in the paediatric population mention clinical studies to evaluate food effects in any capacity [7].

## 3. Paediatric Diet and Composition of Food

The composition of meals is subject to huge regional variation. The composition of a meal may not only affect the intestinal physiology, it can also affect the solubility of a drug. Typically a high fat meal will assist in the solubilisation of a poorly water soluble drug; this increased solubility provides a higher concentration gradient to drive absorption of a drug. For example, the bioavailability (in adults) of the poorly soluble drug atovaquone (log P 5.07) was unchanged by intake of two slices of toast, but increased 3- to 3.9-fold by further administration of 28 and 56 g of butter, respectively [8]. Similarly, the bioavailability (in adults) of griseofulvin (log P 1.9) was unchanged by intake of protein-rich or carbohydrate-rich, low-fat meals, but increased approximately seven times by intake of a lipid-rich meal [9]. However, Finkel *et al*. [10] reported that the absorption of penicillin V was reduced (in children) in the presence of food and also in an oily liquid compared to an aqueous liquid which suggests that the drug is preferentially sequestering within the fat and the overall absorption is reduced.

The composition of meals in paediatric populations differs from that used in adults for many reasons, one is that nutritional needs and recommendations vary with age. Calories are consumed in the form of fat, carbohydrate and protein; fat accounts for approximately 50% of the energy in breast milk and is the main source of energy for infants less than six months old. As an infant matures the proportion of fat is gradually overtaken by carbohydrate as the chief energy source. In terms of unit weight the normal infant has much higher intake from fat and carbohydrate compared to an adult.

### 3.1. Regulatory Advice on Meal Composition

The type of food used within a clinical study to measure a food effect is detailed within regulatory guidance documents. The standardised FDA breakfast was proposed in 2002 [4] and describes a high-fat meal containing 50% to 65% of energy from lipids, 25% to 30% from carbohydrates, and 15% to 20% from proteins, with the meal providing a total of 800 to 1000 kcal. This FDA example high-fat test meal outlined in the guidance document was used to provide the greatest effects on gastro-intestinal (GI) physiology as a lipid-rich meal will be retained longer in the stomach and more bile and pancreatic juice will be secreted so that systemic drug availability would be maximally affected.

A possible composition of the meal would be two slices of toast with butter, two eggs fried in butter, two strips of bacon, 4 oz of hash brown potatoes, and 8 oz of whole milk. European guidance details a standardised high fat meal which is equivalent to the FDA breakfast; although a moderate meal is also listed as one that contains a total of 400–500 kcal of which approximately 150 kcal is fat [4].

EMA guidance highlights the relevance of the composition of the food used within a clinical study in terms of paediatric populations particularly for newborns and young infants where they are fed a predominantly (or exclusively) milk diet [4]. EMA ICH E11 guidance [6] states that for a medicinal product studied in paediatric patients extrinsic factors (e.g., diet) could impact on extrapolation of this data to other geographical regions (due to dietary differences). There has been a recent EMA concept paper on extrapolation of efficacy and safety in medicine development with reference to extrapolation of data from adults into paediatric populations and also across age subsets within the paediatric population, however there is no direct mention of extrapolation of food effects [11].

Milk has a similar composition to a standard FDA breakfast with respect to the ratio of carbohydrate to fat to protein [12]. The meal used in paediatric populations varies due to the lack of regulatory guidance; in younger children milk is commonly used whereas a breakfast is used in older children. There is often debate about the relevance of the meal used in terms of mimicking a typical eating pattern in the relevant population. However, it is important that the meal used provides the maximum effect on gastrointestinal physiology aligned to the adult guidance; therefore a milk meal or portion size controlled FDA standard breakfast should form the meal of choice in such clinical studies.

### 3.2. Paediatric Feeding Patterns

**Figure 1:**
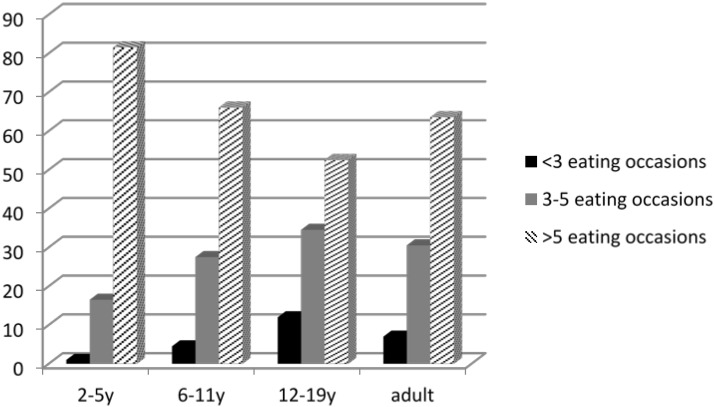
Influence of age on number of daily eating occasions within the United States of America (data extracted from [13]).

Typical Western adult eating patterns are considered to comprise of three meals per day; although there is data to suggest that this is increasing with the total number of eating occasions being greater than 5 in over 60% of American adults [13]. If it is assumed that an adult sleeps for 8 h; the likely time interval between eating occasions is likely to be less than 4 h. A typical newborn will feed approximately 10 times daily [14], although this varies according to culture and global region. The pattern of eating occasions with age is shown in Figure 1 below.

Within the regulatory guidance for conducting food effect clinical studies, the standardised breakfast meal is to be ingested after an overnight fast of at least 10 h. The meal should be eaten in 30 min or less, and at 30 min the drug product should be administered together with 240 mL of water. One hour after administration, water ad libitum is allowed and four hours post-dose food is allowed [4]. There is no similar guidance on the conduct of food effect studies in children although direct translation of this methodology to children younger than two years may lead to non-compliance both in terms of fasting periods and consumption of 240 mL water. It should also be noted that this practice is unlikely to represent typical habits in all populations in general practice.

### 3.3. Volume of Food Consumed

The FDA standard breakfast is reported to have a volume of 513 mL, which is consistent with typical meal volumes in adults [15]. The volume of feed to give to neonates and young infants is typically calculated as 200 mL/kg/day; this equates to a volume of about 90 mL per feed for a term neonate (assuming a 3.5 kg baby fed eight times); up to 190 mL per feed for a six month old infant at 7.6 kg. Functional gastric capacity has been estimated at 30 g/kg body weight; this equates to masses of 250, 285 and 345 g/meal for infants of average body weight at 7, 10 and 12–23 months respectively [16]. The relationship between functional gastric capacity and body weight is not valid for all weights as this would equate to a 75 kg adult requiring a meal of 2250 g, which is much greater than a typical meal. A recommended meal for a school child aged 5–7 has a total mass of 280 g, which assuming a density of close to 1 provides a volume estimate of 280 mL [17]. Therefore it can be assumed that the volume of food increases with age although the relationship is not easily described by a single study. The volume of food consumed per kilogram of bodyweight is higher in younger children; this is likely to be linked to the growth phase of the child.

The amount of food that is necessary to initiate the fed state is not very well elucidated, however, administration of a small amount, 2 g, of long chain MAG to adults was observed to delay gastric emptying [18]. Therefore it is likely that there is greater alteration in gastric emptying in younger children where a greater proportion of the calorific intake is derived from fat. This suggests that volume is less critical than composition in terms of food–drug interactions within paediatric populations.

## 4. Physiological and Anatomical Differences in Paediatric Populations that Affect Drug–Food Interactions

Differences in gastrointestinal physiology and anatomy between paediatric and adult populations can significantly affect the rate of absorption and bioavailability of drugs. There are several excellent reviews that address this topic (e.g., [19]). This review considers these differences with a specific focus on food–drug interactions.

### 4.1. Gastric Emptying

Gastric emptying (GE) determines the onset of absorption as this is a rate-limiting step prior to drug exposure to the absorbing membrane of the small intestine. Many factors affect GE including meal volume and composition. The primary factor that dictates the emptying rate of the stomach is the calorific profile of the contents, but it is also influenced by other factors including volume, osmolality, viscosity, and temperature. Drugs where GE is known to be rate limiting include paracetamol [20]; busulfan [21]; ampicillin [22]; riboflavin [23] and levetiracetam [24]; differences in paediatric meal composition may therefore affect the pharmacokinetic profile of these drugs. There have also been several studies on gastric emptying of breast milk compared to formula and also the effect of fortifiers within breast milk; these show that breast milk empties faster than formula or cows’ milk [25] and that fortifiers do not alter rates of gastric emptying [26].

#### 4.1.1. Calorific Content and GE

The caloric output from the stomach has been found to be between 2 and 4 kcal/min (8.4 and 16.4 kJ/min) [1]. The fat content of a meal plays a critical role in determining the gastric emptying rate as fat contains twice as many calories as carbohydrate [27]. For example, administration of a lipid-rich meal with 200 mL of water resulted in a lag time of gastric emptying of 44 ± 20 min, compared with a lag time of 14 ± 11 min after intake of 200 mL water [28]. The higher the calorific content of the meal the longer the delay to GE and consequently delay to drug absorption.

#### 4.1.2. Volume and GE

Gastric emptying of liquids is related to volume with increased volume showing rapid gastric emptying [29]. A separate study demonstrated that an increase in the volume of the solid component of a mixed solid/liquid meal results in more rapid gastric emptying of the solid but slower liquid emptying [30]. The reduced volume ingested by younger patients may not recreate the emptying rate anticipated by adult studies.

#### 4.1.3. Osmolality and GE

Hexose sugars were previously shown to slow GE in an osmolality-dependent fashion [31]. However, more recent studies indicate that the slowing of GE is more likely to be dependent upon their specific molecular identity [32].

#### 4.1.4. Viscosity and GE

Gastric emptying time is linked to viscosity as solid particles take longer to empty compared to liquids, therefore a viscous meal shows a longer emptying time and thus a delayed Tmax [33]. Although neonates are fed predominantly liquid meals the introduction of soft, semi-solid foods occurs at around 4–6 months in Europe with a progression to more solid foods including bread and pasta at around 8–9 months of age [34]. The viscosity of food is likely to increase with age up to approximately two years and then be consistent through to adulthood; therefore GE may be faster than anticipated in children under two years of age.

#### 4.1.5. Temperature and GE

There have been several studies conducted that examine the impact of temperature on GE that show conflicting results. Sun *et al*. (1988) reported that a liquid meal at body temperature was emptied from the stomach more rapidly that one either colder or warmer; this is of interest for neonates and infants who feed at approximately 37 °C [35]. However, Mishima *et al*., (2009) reported that a hot solid meal (60 °C) significantly accelerates gastric emptying compared to meals at 37 °C and 4 °C [36]. The temperature of food may need to be considered in relation to gastric emptying as well as for potential degradation on the drug product.

### 4.2. Gastrointestinal Transit Times

The transit time within the small intestine is considered to be unaffected by food and estimated as 3–4 h in adults [1]. However, food ingestion typically triggers distal intestine emptying therefore the timing of food in relation to medicines administration can be important. For example, when a non-disintegrating tablet was administered 45 min prior to a standard breakfast, the small intestinal transit time was reduced to approximately 100 min in those subjects where the tablet had already entered the small intestine before the breakfast was given [37]. This has particular relevance in the youngest patients where feeding is more frequent than in adults.

### 4.3. Splanchnic Blood Flow

Food intake induces an increased splanchnic blood flow, which in turn will increase the absorption and transfer of nutrients into the bloodstream. In terms of drug absorption increased splanchnic blood flow will increase the rate that the drug reaches the liver and may lead to alterations in the fraction of drug undergoing first pass metabolism.

The effect of breast milk, adapted cow’s milk formula, and nucleotide supplemented cow’s milk formula on intestinal blood flow in neonates showed that there was no significant difference between the breast milk and adapted cow’s milk formula-fed groups; however, the nucleotide supplemented cow’s milk formula-fed group had significantly higher postprandial blood flow velocity and volume flow. A higher splanchnic blood flow may result in reduced bioavailability of a drug due to greater loss in first pass metabolism.

### 4.4. Membrane Interactions

Drug absorption involves transporters and enzymes which may not be fully mature in paediatric patients. However, the maturation patterns of these moieties are not well understood. Typically, it is reported that maturation follows that for functionality of the digestive system [38]. However, there is some evidence that the ontogeny is affected by feeding, where formula feeding appeared to accelerate maturation of caffeine and dextromethorphan metabolism by increasing the activity of CYP1A2 and CYP3A4, respectively compared to breast feeding [39]. Several plant-derived food and drink components have been shown to modulate enzymes and transporters in the intestine, leading to altered absorption of certain drugs. Commonly consumed fruit juices, teas, and alcoholic drinks contain phytochemicals that inhibit intestinal cytochrome P450 and phase II conjugation enzymes, as well as uptake and efflux transport proteins. Specific studies have shown that CYP 3A4 activity is reduced in the young and reach adult values during adolescence although the data is not conclusive [40]. Examples of foods that contain inhibitors of cytochrome P450 3A4 are grapefruit juice, garlic, and red wine [41]; these have predominantly been explored based on adult diets and there is limited information on paediatric food components. The difference in CYP3A4 expression coupled with consumption of different dietary components may have effects on drug–food interactions observed.

### 4.5. Review of Food Effects in Paediatric Populations

Very few food effect studies have been conducted in paediatric patient populations; those reported in the literature are shown in Table 1. There is much more variability in the meal used to administer medicine doses to children which is most likely related to the lack of clear guidance in this area; the most common foods included milk or standardised breakfasts.

The data in Table 1 show that in total there were 10 incidences where the pharmacokinetic parameters were unchanged in a paediatric population in the presence of food and 11 incidences where food led to a change in PK parameters. Where food altered the bioavailability of the drug the recommendations regarding concomitant administration with food made as a result of the study are included. Tmax was prolonged in many cases with Cmax and AUC being reduced more frequently than increased as a consequence of administration with food. The preceding sections of this review highlighted that for paediatric patients gastric emptying is likely to be slower in the presence of food, so the results are not surprising. The meals utilised when undertaking clinical fed effect studies in paediatric populations are also listed in Table 1. The meals varied in terms of composition, viscosity, volume and hydration such that the variability in composition may influence the results observed to a large extent.

Of the 18 studies reported 11 showed the same pharmacokinetic result as that in adults in a food study; five showed different results to the adult study and two could not be compared. This difference in effects observed in adults compared to paediatric populations is a cause for concern as typically the effects of a food study are extrapolated from an adult population into a paediatric population. This presentation of the data emphasises the risks associated with this approach.

**Table 1:** Food effect studies conducted in paediatric populations.

Drug	Formulation	Dose	Age Range	Meal	Change in Parameter	Comments	Ref	Comparable Adult Data	Adult Ref
Amoxicillin	oral suspension	15 or 25 mg/kg	4 m–45 m (mean 27 months)	4oz milk or formula (Similac or Infamil)	Cmax decreased at 15mg/kg dosing Cmax unchanged at 25 mg/kg dosing AUC unchanged all doses		[42]	Adult study at 500mg dose showed no impact of food (standard breakfast)	[43]
Ampicillin	oral suspension	15 or 25 mg/kg	4 m–45 m (mean 27 months)	4oz milk or formula (Similac or Infamil)	Cmax unchanged AUC unchanged		[42]	Adult study at 500mg dose showed reduction in Cmax and AUC with food (standard breakfast)	[43]
Cefpodoxime proxetil	oral suspension	10 mg/kg	5 m–12 y	Age-appropriate meal (volume and composition)	Tmax prolonged Cmax unchanged	Cefpodoxime can be administered without regard for food	[44]	Cmax and AUC eleveated in the fed states for all meals (high protein, low protein, high fat, low fat).	[45]
Cephalexin	suspension and capsule	25 mg/kg	3–14 y	Standard hospital meal	Cmax reduced (not significant) AUC increased (not significant)	Concomitant administration of food does not substantially affect absorption	[46]	Absorption is delayed but AUC is not appreciably altered	[47]
Clarithromycin	oral suspension	7.5 mg/kg			AUC unchanged		[48]	The extent of absorption is relatively unaffected by the presence of food	[49]
Desmopressin	oral lyophilisate (MELT)	120 mg	mean age 12.7 y	Standardised meal	Cmax unchanged AUC unchanged	Bioequivalence established, even with concomitant food-intake	[50]	AUC and Cmax are reduced with food in adults (for the tablet formulation but not the MELT)	[51]
Didanosine		50 or 150 mg/m^2^			Cmax reduced AUC unchanged	Take in the fasted state	[52]	AUC and Cmax are substantially reduced with some formulations if taken with food	[53]
Griseofulvin	oral suspension	10 or 15 mg/kg/day	19 m–11 y (mean 4.8 y)	120 mL whole milk	Cmax increased AUC increased	Drug should be administered with whole milk or other food containing fat for optimum bioavailability	[54]	Cmax increased AUC increased	[55]
Lumefantrine	dispersible or crushed tablets (Coartem ^®^) with 10mL water		0.25–12.4 y	Categorised as: none; breast feeding; liquid (soup, broth); pancake; porridge or other	Cmax increased (greater increased for crushed tablet) Pancake increased the exposure to a greater extent than milk	Consumption of food at the time of dosing remains advisable	[56]	Cmax and AUC increased when given with food	[57]
6-mercaptopurine		75 mg/m2		250 mL milk and 50 g biscuits	Tmax prolonged Cmax significantly reduced AUC significantly reduced	6-MP should be taken in a fasting state to optimize drug absorption in children undergoing chemotherapy for ALL	[58]	Tmax prolonged Cmax reduced	[59]
6-mercaptopurine			4 year old child (*n* = 1)	Milk or fruit squash	In the presence of milk Cmax reduced AUC reduced	Child required increased dose of mercaptopurine when taken with milk	[60]	Tmax prolonged Cmax reduced	[59]
6-mercaptopurine				Breakfast (milk or yogurt plus cereal, or sandwiches)	Cmax unchanged AUC unchanged	Insufficient data for a recommendation	[61]	Tmax prolonged Cmax reduced	[59]
Methotrexate		15 mg/m^2^	3–15 years	Milky meal = milk, cornflakes sugar, bread and butter Or citrus meal = orange juice, fresh orange, bread, butter and jam	Milky meal: Tmax prolonged Cmax and AUC significantly reduced Citrus meal: Cmax and AUC unchanged	Methotrexate absorption is delayed by food, particularly milk. For maximum absorption methotrexate should **not** be taken at meal times.	[62]	Tmax prolonged AUC unchanged	[63]
Penicillin V (phenoxymethylpenicillin)	dispersed in water 23 mg/mL (Calciopen)	20 mg/kg	6 m–5 years	Breakfast	Cmax significantly reduced AUC significantly reduced	Dosing Penicillin V with food will reduce its exposure	[10]	Slight alteration in pharmacokinetics such that it is recommended to dose penicillin V in the fasted state	[64]
Penicillin V (phenoxymethylpenicillin)	Suspension		infants and children	milk	Cmax reduced AUC reduced	Dosing Penicillin V with milk will reduce its exposure	[65]	Slight alteration in pharmacokinetics such that it is recommended to dose penicillin V in the fasted state	[64]
Propylthiouracil	Not stated	100–280 mg/m^2^		Not stated	Tmax prolonged Cmax reduced AUC variable	Propylthiouracil administration in the fasting state is advisable	[66]	Cmax unchanged AUC unchanged	[67]
Theophylline	slow release products			Standard breakfast of cornflakes, rye bread, butter, salami and low fat milk	Effects dependent upon formulation	Food effect is dependent upon formulation. Caution advised if switching brand.	[68]	Food has substantial but variable effects on absorption from modified-release formulations in adults.	[69]
6-thioguanine	Not stated	40 mg/m^2^	1–16 years	Standard breakfast of cereal with milk, toast and a glass of milk	Tmax prolonged Cmax significantly reduced AUC significantly reduced	Although there is a reduction in exposure with food there was no difference in 6-TGN concentrations after 4 weeks. Taking the drug on an empty stomach may not be necessary.	[70]		

The difference in food-effects between adult and paediatric populations is highlighted by a study on four different sustained-release theophylline formulations where the food effect was more marked in children compared to adults; the bioavailability in the fed state was reduced from 100% to 24% in children compared to a reduction to 67% in adults [68].

## 5. Physico-Chemical Food–Drug Interactions

Classification of food–drug interactions aids in the prediction and prevention of their occurrence. Interactions between food and drug within the environmental matrix of the gastro-intestinal tract are usually physico-chemical rather than physiological or anatomical. These often relate to the formulation as well as the drug. The different composition of food consumed by paediatric populations may alter the incidence of this type of interaction.

### 5.1. pH Effects

The absorption of weak acids and bases changes in the presence of food due to the impact of food on the pH of the gastric environment. Typically weak bases can show reduced dissolution and therefore absorption in the presence of food due to the increased gastric pH (e.g., isoniazid [71] and indinavir [72]). Gastric pH in neonates is known to be somewhat higher than adult values therefore the presence of food may not further affect the absorption of weakly basic drugs. Conversely weak acids, including ibuprofen, have shown increased absorption in the presence of food which may also be attributed to a pH effect [73]. Typically a paediatric diet is likely to be of a similar pH to that of an adult; however additional caution is required when dosing with fruit juices which are known to have a low pH.

The composition of food stuffs used to mix with drugs to aid administration also needs careful consideration for weakly acidic or basic drugs. Typically fruit juices are used or applesauce; these have pH values in the region of 3.3–4.1 [74]. Co-administration may therefore impede the absorption of weakly acidic drugs or enhance the absorption of weak bases. The stability of drugs co-mixed with food or drinks has previously been investigated (e.g., [75]).

### 5.2. Viscosity

A high viscosity within the intestinal lumen can reduce the diffusion rate of a drug and therefore reduce its overall absorption; this has previously been shown with bidisomide [76] in adults and with paracetamol, mefenamic acid, hydrochlorothiazide and cimetidine in dogs [77]. The composition of the meal is therefore paramount in understanding the viscosity within the gastro-intestinal lumen.

In children younger than two years the overall absorption of certain drugs (e.g., bidisomide) may be increased due to the lower relative viscosity of the ingested food.

### 5.3. Binding/Chelation

Chelation or binding of drugs to components within the gastrointestinal lumen can reduce their absorption. For example, ciprofloxacin bioavailability is significantly reduced as a result of chelation in the presence of enteral nutrition formula [78]. Chelation to metal cations is the most widely reported food interaction of this type that limits absorption with fluroquinolones, tetracyclines and some oral cephalosporins.

Many cereals and fruit juices targeted at children are fortified with additional calcium, iron, magnesium and vitamins; this additional level of cations may enhance chelation and reduce bioavailability of drugs susceptible to chelation. The standard FDA breakfast contains 435 mg calcium, 4 mg iron, 72 mg magnesium and 3.2 mg zinc; whereas a breakfast consisting of fortified cereal and calcium-fortified orange juice would contain approximately 1600 mg calcium [79]. This type of food interaction needs special consideration in paediatric populations.

Milk, is a significant component of neonate and infants’ diets yet has been shown to reduce the absorption of many drugs including ketoprofen [80], mercaptopurine [60,81], methotrexate [62] and penicillin [65]. Proteins within food can also bind with drugs to reduce their exposure, for example phenytoin [82]. The composition of food in terms of protein and cations needs to be considered in the design of appropriate tests to predict food–drug interactions in children.

### 5.4. Thermal Degradation

Many drugs degrade at higher temperatures; the rate of degradation is proportional to the temperature as described by the Arrhenius equation. Exposure to high temperatures can also degrade other components of a medicine including taste masking excipients. Concomitant administration of drugs with hot drinks and hot meals is well known in adults. Although paediatric patients do not typically drink very hot liquids it is common to prepare formula milk with boiling water and to leave to stand to cool prior to administration. If a drug is added to a bottle at the same stage as the powdered formula, exposure to boiling water and high temperatures for the period of cooling (up to 30 min) could have an adverse effect on the stability of the drug and therefore on drug exposure to the patient.

## 6. Formulation Influence on Food Effects

Liquids are emptied from the stomach faster than solid food items, as the dimension of particles that can pass through the pylorus can limit gastric emptying. Tablet disintegration testing sets a particle size limit of 1.8–2.2 mm [83] as the relevant size to pass through the pyloric sphincter in adults as larger particles (>2 mm) require Phase III of the migrating motor complex (MMC) for ejection from the stomach [84]. The equivalent size is yet to be evaluated in children. When liquid medicines are administered in the presence of food their transit to the small intestine may be delayed and drug exposure profile altered compared to dosing in the fasted state [85]. The presence of food can also influence the disintegration of tablets due to the different mixing forces and consequently the time taken for sufficiently small tablet particles to transfer to the small intestine. Clinical studies have demonstrated a delay in absorption of drugs due to prolonged disintegration times in the fed state in dogs [86] and in man [87].

Formulation factors have previously been shown to affect onset of action and absorption and paediatric paracetamol suspensions have been designed to provide a rapid onset of action in both the fasted and fed state to reduce variability in pharmacokinetics [88]. The formulation used can affect the food effect observed therefore caution may be required when switching formulations in terms of instructions for administration with food. In paediatric patients where drugs are more frequently administered as solutions, suspensions or extemporaneously crushed tablets this disintegration step has been removed; therefore the food effect may be different to that predicted from adult studies so extra vigilance is required.

Milk-based formulations have been developed specifically to enhance the bioavailability of certain poorly soluble drugs [89]; these formulations have been shown to be stable and demonstrate that milk can be used as a component of the formulation as well independently to aid in drug administration [90].

### 6.1. Review of Food Effects in Paediatric Formulations

Pharmacokinetic data and bioequivalence studies are typically conducted in adult populations for formulation changes, including the development of paediatric formulations. Table 2 details the existing published data on fed effect studies undertaken on formulations developed for paediatric use.

**Table 2:** Food effect studies on paediatric formulations conducted in adult populations.

Drug	Formulation	Dose	Food/Meal	Timing of Dose	Change in Parameter	Remarks from Reference	Reference
Clobazam	crushed tablet	20 mg	Applesauce		Cmax unchanged AUC unchanged	Clobazam tablets can be given crushed with applesauce. Administration of clobazam with a high-fat meal did not affect clobazam exposure	[91]
Lansoprazole	enteric coated granules (capsule contents)	30 mg	1 tablespoon of yogurt or 1 tablespoon Ensure^®^ pudding or 1 tablespoon Cottage cheese	overnight fast —3 h post dose	Tmax prolonged (for cottage cheese) Cmax unchanged AUC unchanged	The bioavailability when administered in yogurt, Ensure^®^ pudding and cottage cheese, was similar to that of the intact capsule in these healthy adult volunteers	[92]
Lansoprazole	enteric coated granules (capsule contents)	30 mg	180mL orange juice without pulp or tomato juice; **or** soft food (1 tablespoon of strained pears)	overnight fast —3 h post dose	Tmax prolonged (for orange juice only) Cmax unchanged AUC unchanged	The bioavailability when administered in orange juice, tomato juice, or a small amount of strained pears, was similar to that of the intact capsule in these healthy adult volunteers	[93]
Levetircetam	crushed tablet	500 mg	4oz applesauce 120 mL enteral nutrition formula (Sustacal^®^)	overnight fast —4 h post dose	Cmax unchanged AUC unchanged	The overall rate and extent of absorption was not significantly impaired after crushing and mixing of the tablet with either a food vehicle or a typical ENF product	[94]
Methylphenidate	extended release granules (capsule contents)	20 mg	1 level tablespoon applesauce (15 mL)	No data available	Cmax unchanged AUC unchanged	The bioavailability of methylphenidate was not altered by sprinkling their contents onto a small amount of applesauce	[95]
Morphine	extended release granules (capsule contents)	60 mg	2 tablespoons applesauce	overnight fast —4 h post dose	Cmax unchanged AUC unchanged	The bioavailability when sprinkled onto applesauce was similar to that of the intact capsule in adults	[96]
Rabeprazole	enteric coated granules (capsule contents)	10 mg	1 tablespoon of yogurt 1 tablespoon applesauce 5mL infant formula	overnight fast —4 h post dose	Cmax unchanged AUC unchanged	The bioavailability of rabeprazole granules was similar for all food stuffs evaluated	[97]
Azithromycin	paediatric suspension (cherry-banana) (40mg/mL)	500 mg	High fat breakfast and 227 mL of whole milk, ingested within a 20 min period	overnight fast —4 h post dose	Cmax increased AUC unchanged	The suspension formulation may be administered without regard to meals, increasing the convenience of once-daily dosing regimens	[98]
Everolimus	dispersible tablet	1.5 mg	Standardized, high-fat breakfast	overnight fast —4 h post dose	Tmax prolonged Cmax reduced AUC unchanged	Administer the dispersible tablet to each patient on a consistent basis either with or without food.	[99]
Glycopyrrolate	oral solution (1 mg/5mL)	2 mg	FDA High fat breakfast	overnight fast —4 h post dose	Cmax reduced AUC reduced	Administer at least one hour before or after meals	[100]
Ibuprofen	chewable tablets	200 mg	Standardised breakfast plus 240 mL whole milk total calorie content = 650 calories	overnight fast —4 h post dose	Tmax prolonged Cmax reduced AUC slightly decreased		[101]
Nelfinavir	powder to mix with food	100–800 mg	Standardised breakfast	overnight fast —4 h post dose	Tmax prolonged Cmax increased AUC increased	Recommended that patients take nelfinavir with a meal or snack	[102]
Nitazoxanide	Suspension (25 mL of 100 mg/5 mL)	500 mg	High fat high calorie standardised breakfast and 240 mL whole milk	overnight fast —4 h post dose	Tmax prolonged Cmax unchanged AUC increased		[103]
Paracetamol	Suspension (42 mL of 24 mg/mL )	1008 mg	Light calorie low fat breakfast	meal 2.5 h prior to dosing (semi-fed state)	Tmax prolonged Cmax unchangedAUC unchanged	Food had a significant effect on the early exposure and onset of therapeutic level of paracetamol from the paediatric suspension	[88]
Ritonavir	oral solution	600 mg	514 KCal; 9% fat, 12% protein and 79% carbohydrate)		Tmax prolonged Cmax decreased AUC decreased		[104]
Rufinamide	oral suspension (40 mg/mL)	400 mg	High fat meal	overnight fast—4 h post dose	Tmax prolonged Cmax increased AUC increased	The rufinamide suspension is bioequivalent to the approved tablets	[105]
Sertraline HCl	oral solution	100 mg	Standardised breakfast	overnight fast—4 h post dose	Cmax unchanged AUC unchanged	The pharmacokinetics of the sertraline oral solution are similar under fed and fasted conditions	[106]
Topiramate	oral solution (20 mL 5mg/mL)	100 mg	High fat low calorie meal	overnight fast—4 h post dose	Tmax prolonged Cmax unchanged AUC unchanged	A high-fat, high-calorie meal delays absorption of liquid topiramate without changing overall topiramate exposure when compared to fasted conditions.	[107]
Tripanavir	oral solution 100 mg/mL (co administered with 200 mg ritonavir)	500 mg of			Cmax reduced slightly AUC unchanged	Oral solution can be administered to patients either with or without food. The current label recommends the tipranavir capsules be taken with food.	[108]

The data presented shows a clear division into studies conducted that measure the effect of mixing medicines with the recommended food for administration (e.g., applesauce, yogurt infant formula) and those undertaken to understand a broader food effect. Supporting studies that measure the degradation of drugs in soft food to predict any *ex vivo* impact of food manipulation on drug stability have been conducted (e.g., [109]) although these are outside the scope of this review.

As expected, all studies conducted using the recommended food for administration showed no significant change in bioavailability compared to the fasted state. However, the extrapolation of these studies into paediatric patients may be more complex than anticipated; particularly when considering the volume of food administered: a tablespoon in all studies [92,93,94,95,96,97] followed by 120–240 mL (median 180 mL) of water which may not be a representative ratio in younger children. A reduced volume of both food and drink may have consequences on absorption resulting in a difference in the pharmacokinetic profile compared to fasted administration.

It may also be necessary to consider the nature of the food utilised in common practice as the recommended food (e.g., applesauce) may be substituted for yogurt, jam or other foodstuffs resulting in a different pharmacokinetic profile [2]. The impact of a change in diet on pharmacokinetics has previously been reported; for example milk appeared to have a greater effect on 6-mercaptopurine compared to fruit juice [60].

The data in Table 2 shows that a standardised breakfast was used in most studies where a broader food effect was investigated. Of these 12 food effect studies, three demonstrated no pharmacokinetic impact of food and nine showed a clear impact with food. Where a food effect was reported this was managed by appropriate patient information labelling, for example, Kovarik *et al*., (2003) [99] compared the pharmacokinetic profile of everolimus as conventional IR tablets and a dispersible paediatric formulation in adults, as well as the effect of food on the absorption of the paediatric formulation. The result showed that the Cmax was halved in the presence of food yet the AUC remained the same therefore providing that the patient is consistent in how they take the drug their therapy is not compromised [99]. Other drugs have a therapeutic index more closely linked to the Cmax and in such cases the food effect may be more significant and require additional management.

## 7. Predicting a Food Effect

The effect of food on the pharmacokinetic profile of a drug is a significant concern during drug development and within subsequent clinical practice. A system that enables prediction of a food effect during development would have many benefits. However, it is recognised that such a model is complex due to the concurrent ongoing chemical and physiological variables associated with the postprandial changes in the gastrointestinal tract. This section highlights existing methods used to predict food effects for adult populations and explores their extrapolation into paediatric populations.

### 7.1. Theoretical Models

Previous studies [110] have linked physicochemical properties of drugs to the likelihood of a food effect in adult populations. Typical drug physicochemical properties included the dose:solubility ratio and drug lipophilicity which are the foundations of the biopharmaceutical classification system [111]. Key differences in paediatric patients’ physiology and anatomy as well as dose adjustments are likely to result in differences in solubility values used in prediction of a food effect in adults. Therefore it is likely that these methods may not be appropriate in the prediction of food effects in paediatric patients. Typically intestinal concentrations and the dose:solubility ratio are likely to be higher in the youngest paediatric patients due to the lower volumes of intestinal fluids; resulting in a greater likelihood of a food effect for poorly soluble drugs.

### 7.2. Physical Models

#### 7.2.1. Solubility/Dissolution Testing

Alternative methods used to predict food effects include *in vitro* dissolution testing where the dissolution media is representative of adult fed gastrointestinal fluids. Fed state simulated intestinal media (FeSSIF) was developed to mimic intestinal fluid in the fed state and is currently the media of choice in predicting fed effects in adults [112]. Previous attempts to simulate typical postprandial gastric conditions include homogenised meals including the FDA standard breakfast, emulsions and complete nutrition products [113]. The current most appropriate simulated fluids that mimic the fed conditions within the stomach (in adults) are milk and Ensure^®^ Plus (Ensure Plus^®^ is a complete nutrition product) [12]. Both standardized homogenized cows’ milk with a fat content of 3.5% (whole milk) and Ensure^®^ Plus have a similar composition to the FDA standard breakfast meal with respect to the ratio of carbohydrate/fat/protein [15]. Obviously milk has benefits in terms of simulating the gastric contents in neonates and young infants.

The viscosity of the standard FDA breakfast was measured by Klein *et al*., (2004); this was greater than both milk, Ensure and Ensure plus^®^ (nutrition drinks). In order to attain a viscosity similar to that of a solid meal a thickening agent was required to be added to the liquid media to appropriately mimic the physical properties of the fed state [15]. However, none of these media reflects all parameters that are important for determining food effects on drug release in the stomach (specifically in terms of pH changes and pepsin concentration).

Dissolution testing using FeSSIF has previously shown good correlation to adult *in vivo* data, particularly for poorly soluble drugs (e.g., [114]). A technique that combined dissolution apparatus with a caco-2 monolayer to measure drug permeation was reported by Kataoka *et al*., (2006) [115]. This technique has provided *in vitro* data that correlated well to *in vivo* data for several drugs administered in the fed state. Other dynamic dissolution models that mimic the gastrointestinal tract in the fed state include TNO testing [116] and the dynamic gastric model [117]. A bespoke dynamic dissolution apparatus mimicking the conditions in the upper gastrointestinal tract of neonates, infants and toddlers has been reported where food effects were evaluated *in vitro* [118]. However, extrapolation of biorelevant dissolution testing to provide correlations to *in vivo* paediatric fed state data is yet to be reported.

### 7.3. *In Silico* Models

Physiologically based pharmacokinetic models have become a widely used tool for predicting food effects and simulating plasma profiles of drugs, particularly poorly soluble drugs. Typically these models rely on input parameters that include the solubility of the drug in both fasted and fed media as well as dissolution data from FeSSIF media. However, their advantage over simple dissolution or solubility testing lies in the incorporation of permeability data and can allow the pattern of intestinal transit to be altered to match the fed state in adults. This methodology can be used to generate *in vivo* *in silico* relationships to better understand and predict food effects [119]. Although several physiologically relevant paediatric pharmacokinetic models are available (e.g., SimCYP, Gastroplus) there is no data on prediction of food effects within paediatric populations using these models [120].

## 8. Conclusions

Although the practice of mixing medicines with food to enhance acceptability to children is known to be widespread, the consequences are unexplored for many drugs.

The lack of regulation surrounding fed studies conducted in children and young people leads to great variability in the design and conduct of such studies. This leads to confusion as to what foods are and are not acceptable for co-administration with a particular medicine with potentially serious consequences. Additional research is required to better understand which foods should be used to provide a worst-case scenario equivalent to the FDA breakfast that is used in adult fed effect studies.

Fed effects observed in adult populations are not necessarily observed within a paediatric population and *vice versa*; therefore extrapolation of such effects needs to be undertaken with caution. The restricted availability of pharmacokinetic data from fed-paediatric clinical studies currently limits the development of relevant and validated *in vitro* and *in silico* tests to better predict food effects within paediatric populations. There is insufficient evidence to justify extrapolation of existing methods used to predict food effects in adults directly to paediatric populations.

In conclusion, the impact of food on the pharmacokinetics in children cannot be predicted using existing methods. Additional research is required to understand the physiological and anatomical factors that can influence the absorption of a drug in paediatric populations. Furthermore, food effect studies should be undertaken in paediatric patients where a known food effect occurs in adults.

## References

[1] Won C.S., Oberlies N.H., Paine M.F. (2012). Mechanisms underlying food-drug interactions: Inhibition of intestinal metabolism and transport. Pharmacol. Ther..

[2] Akram G., Mullen A.B. (2012). Paediatric nurses’ knowledge and practice of mixing medication into foodstuff. Int. J. Pharm. Pract..

[3] (2012). BNF for Children 2011–2012. http://www.pharmpress.com/product/9780853699590/bnfc.

[4] FDA (2002). Guidance for Industry: Food-Effect Bioavailability and Fed Bioequivalence Studies.

[5] FDA (2003). Guidance for Industry: Exposure-Response Relationships—Study Design, Data Analysis and Regulatory Applications.

[6] EMA (2011). ICH Topic E11. Clinical Investigation of Medicineal Products in the Paediatric Population. CPMP/ICH/2711/99.

[7] EMA (2006). Guideline on the role of pharmacokinetics in the development of medicinal products in the paediatric population. EMEA/CHMP/EWP/147013/2004.

[8] Rolan P.E., Mercer A.J., Weatherley B.C., Holdich T., Meire H., Peck R.W., Ridout G., Posner J. (1994). Examination of some factors responsible for a food-induced increase in absorption of atovaquone. Br. J. Clin. Pharmacol..

[9] Crounse R.G. (1961). Human pharmacology of griseofulvin: The effect of fat intake on gastrointestinal absorption. J. Investig. Dermatol..

[10] Finkel Y., Bolme P., Eriksson M. (1981). The effect of food on the oral absorption of penicillin V preparations in children. Acta Pharmacol. Toxicol. (Copenh).

[11] EMA (2012). Concept Paper on Extrapolation of Efficacy and Safety in Medicine Development.

[12] Klein S., Butler J., Hempenstall J.M., Reppas C., Dressman J.B. (2004). Media to simulate the postprandial stomach I. Matching the physicochemical characteristics of standard breakfasts. J. Pharm. Pharmacol..

[13] US Department of Agriculture Meals and Snacks: Distribution of Meal Patterns and Snack Occasions, by Gender and Age, *What We Eat in America NHANES*
**2009–2010**. http://www.ars.usda.gov/SP2UserFiles/Place/12355000/pdf/0910/Table_33_DMP_GEN_09.pdf.

[14] WHO Working Group on the Growth Reference Protocol, WHO Task Force on Methods for the Natural Regulation of Fertility (2002). Growth of healthy infants and the timing, type, and frequency of complementary foods. Am. J. Clin. Nutr..

[15] Klein S., Butler J., Hempenstall J.M., Reppas C., Dressman J.B. (2004). Media to simulate the postprandial stomach I. Matching the physicochemical characteristics of standard breakfasts. J. Pharm. Pharmacol..

[16] WHO Complementary Feeding of Young Children in Developing Countries. http://www.who.int/nutrition/publications/infantfeeding/WHO_NUT_98.1/en/index.html.

[17] Francis D.E.M. (1986). Nutrition for Children.

[18] Kossena G.A., Charman W.N., Wilson C.G., O’Mahony B., Lindsay B., Hempenstall J.M., Davison C.L., Crowley P.J., Porter C.J. (2007). Low dose lipid formulations: effects on gastric emptying and biliary secretion. Pharm. Res..

[19] Kaye J.L. (2011). Review of paediatric gastrointestinal physiology data relevant to oral drug delivery. Int. J. Clin. Pharm..

[20] Levy G., Khanna N.N., Soda D.M., Tsuzuki O., Stern L. (1975). Pharmacokinetics of acetaminophen in the human neonate: Formation of acetaminophen glucuronide and sulfate in relation to plasma bilirubin concentration and D-glucaric acid excretion. Pediatrics.

[21] Hassan M., Ljungman P., Bolme P., Ringden O., Syruckova Z., Bekassy A., Stary J., Wallin I., Kallberg N. (1994). Busulfan bioavailability. Blood.

[22] Silverio J., Poole J.W. (1973). Serum concentrations of ampicillin in newborn infants after oral administration. Pediatrics.

[23] Jusko W.J., Khanna N., Levy G., Stern L., Yaffe S.J. (1970). Riboflavin absorption and excretion in the neonate. Pediatrics.

[24] Toublanc N., Sargentini-Maier M.L., Lacroix B., Jacqmin P., Stockis A. (2008). Retrospective population pharmacokinetic analysis of levetiracetam in children and adolescents with epilepsy: dosing recommendations. Clin. Pharmacokinet..

[25] Garzi A., Messina M., Frati F., Carfagna L., Zagordo L., Belcastro M., Parmiani S., Sensi L., Marcucci F. (2002). An extensively hydrolysed cow’s milk formula improves clinical symptoms of gastroesophageal reflux and reduces the gastric emptying time in infants. Allergol. Immunopathol..

[26] McClure R.J., Newell S.J. (1996). Effect of fortifying breast milk on gastric emptying. Arch. Dis. Child. Fetal Neonatal Ed..

[27] Hunt J.N., Stubbs D.F. (1975). The volume and energy content of meals as determinants of gastric emptying. J. Physiol..

[28] Sunesen V.H., Vedelsdal R., Kristensen H.G., Christrup L., Mullertz A. (2005). Effect of liquid volume and food intake on the absolute bioavailability of danazol, a poorly soluble drug. Eur. J. Pharm. Sci..

[29] Hunt J.N., Smith J.L., Jiang C.L. (1985). Effect of Meal Volume and Energy Density on the Gastric-Emptying of Carbohydrates. Gastroenterology.

[30] Collins P.J., Horowitz M., Maddox A., Myers J.C., Chatterton B.E. (1996). Effects of increasing solid component size of a mixed solid/liquid meal on solid and liquid gastric emptying. Am. J. Physiol..

[31] Meeroff J.C., Go V.L.W., Phillips S.F. (1975). Control of Gastric-Emptying by Osmolality of Duodenal Contents in Man. Gastroenterology.

[32] Little T.J., Gopinath A., Patel E., McGlone A., Lassman D.J., D’amato M., McLaughlin J.T., Thompson D.G. (2010). Gastric emptying of hexose sugars: role of osmolality, molecular structure and the CCK1 receptor. Neurogastroenterol. Motil..

[33] Shimoyama Y., Kusano M., Kawamura O., Zai H., Kuribayashi S., Higuchi T., Nagoshi A., Maeda M., Mori M. (2007). High-viscosity liquid meal accelerates gastric emptying. Neurogastroenterol. Motil..

[34] WHO Feeding and Nutrition of Infants and Young Children. http://www.who.int/nutrition/publications/infantfeeding/9289013540/en/index.html.

[35] Bateman D.N. (1982). Effects of meal temperature and volume on the emptying of liquid from the human stomach. J. Physiol..

[36] Mishima Y., Amano Y., Takahashi Y., Mishima Y., Moriyama N., Miyake T., Ishimura N., Ishihara S., Kinoshita Y. (2009). Gastric emptying of liquid and solid meals at various temperatures. J. Gastroenterol..

[37] Fadda H.M., McConnell E.L., Short M.D., Basit A.W. (2009). Meal-induced acceleration of tablet transit through the human small intestine. Pharm. Res..

[38] Edginton A.N., Fotaki N., Dressman J.B., Reppas C. (2010). Oral Drug Absorption in Pediatric Populations. Oral Drug Absorption: Prediction and Assessment.

[39] Blake M.J., Abdel-Rahman S.M., Pearce R.E., Leeder J.S., Kearns G.L. (2006). Effect of Diet on the Development of Drug Metabolism by Cytochrome P-450 Enzymes in Healthy Infants. Pediatr. Res..

[40] Johnson T.N., Thomson M. (2008). Intestinal metabolism and transport of drugs in children: The effects of age and disease. J. Pediatr. Gastroenterol. Nutr..

[41] Fujita K.-I. (2004). Food-drug interactions via human cytochrome P450 3A (CYP3A). Drug Metab. Drug Interact..

[42] Ginsburg C.M., McCracken G.H., Thomas M.L., Clahsen J. (1979). Comparative pharmacokinetics of amoxicillin and ampicillin in infants and children. Pediatrics.

[43] Eshelman F.N., Spyker D.A. (1978). Pharmacokinetics of amoxicillin and ampicillin: Crossover study of the effect of food. Antimicrob. Agents Chemother..

[44] Kearns G.L., Abdel-Rahman S.M., Jacobs R.F., Wells T.G., Borin M.T. (1998). Cefpodoxime pharmacokinetics in children: effect of food. Pediatr. Infect. Dis. J..

[45] Hughes G.S., Heald D.L., Barker K.B., Patel R.K., Spillers C.R., Watts K.C., Batts D.H., Euler A.R. (1989). The effects of gastric pH and food on the pharmacokinetics of a new oral cephalosporin, cefpodoxime proxetil. Clin. Pharmacol. Ther..

[46] Tetzlaff T.R., McCracken G.H., Thomas M.L. (1978). Bioavailability of cephalexin in children: Relationship to drug formulations and meals. J. Pediatr..

[47] Wise R. (1990). The pharmacokinetics of the oral cephalosporins—A review. J. Antimicrob. Chemother..

[48] Guay D.R., Craft J.C. (1993). Overview of the pharmacology of clarithromycin suspension in children and a comparison with that in adults. Pediatr. Infect. Dis. J..

[49] Rodvold K.A. (1999). Clinical pharmacokinetics of clarithromycin. Clin. Pharmacokinet..

[50] De Guchtenaere A., Hoebeke P., Dehoorne J., Raes A., van Laecke E., Vande Walle J. (2012). 734 Pharmacokinetic Data on Oral Desmopressin Reducing Dosage by Changing to a New Oral Lyophilisate (Melt) Formulation. J. Urol..

[51] Rittig S., Jensen A.R., Jensen K.T., Pedersen E.B. (1998). Effect of food intake on the pharmacokinetics and antidiuretic activity of oral desmopressin (DDAVP) in hydrated normal subjects. Clin. Endocrinol..

[52] Stevens R.C., Rodman J.H., Yong F.H., Carey V., Knupp C.A., Frenkel L.M. (2000). Effect of food and pharmacokinetic variability on didanosine systemic exposure in HIV-infected children. Pediatric AIDS Clinical Trials Group Protocol 144 Study Team. AIDS Res. Hum. Retrovir..

[53] Shyu W.C., Knupp C.A., Pittman K.A., Dunkle L., Barbhaiya R.H. (1991). Food-induced reduction in bioavailability of didanosine. Clin. Pharmacol. Ther..

[54] Ginsburg C.M., McCracken G.H., Petruska M., Olsen K. (1983). Effect of feeding on bioavailability of griseofulvin in children. J. Pediatr..

[55] Ahmed I.S., Aboul-Einien M.H., Mohamed O.H., Farid S.F. (2008). Relative bioavailability of griseofulvin lyophilized dry emulsion tablet *vs.* immediate release tablet: A single-dose, randomized, open-label, six-period, crossover study in healthy adult volunteers in the fasted and fed states. Eur. J. Pharm. Sci..

[56] Borrmann S., Sallas W.M., Machevo S., González R., Björkman A., Mårtensson A., Hamel M., Juma E., Peshu J., Ogutu B. (2010). The effect of food consumption on lumefantrine bioavailability in African children receiving artemether–lumefantrine crushed or dispersible tablets (Coartem^®^) for acute uncomplicated Plasmodium falciparum malaria. Trop. Med. Int. Health.

[57] White N.J., van Vugt M., Ezzet F. (1999). Clinical pharmacokinetics and pharmacodynamics and pharmacodynamics of artemether-lumefantrine. Clin. Pharmacokinet..

[58] Riccardi R., Balis F.M., Ferrara P., Lasorella A., Poplack D.G., Mastrangelo R. (1986). Influence of food intake on bioavailability of oral 6-mercaptopurine in children with acute lymphoblastic leukemia. Pediatr. Hematol. Oncol..

[59] Burton N.K., Barnett M.J., Aherne G.W., Evans J., Douglas I., Lister T.A. (1986). The effect of food on the oral administration of 6-mercaptopurine. Cancer Chemother. Pharmacol..

[60] Sofianou-Katsoulis A., Khakoo G., Kaczmarski R. (2006). Reduction in bioavailability of 6-mercaptopurine on simultaneous administration with cow’s milk. Pediatr. Hematol. Oncol..

[61] Lönnerholm G., Kreuger A., Lindström B., Myrdal U. (1989). Oral Mercaptopurine in Childhood Leukemia: Influence of Food Intake on Bioavailability. Pediatr. Hematol. Oncol..

[62] Pinkerton C.R., Glasgow J.F.T., Welshman S.G., Bridges J.M. (1980). Can food influence the absorption of methotrexate in children with acute lymphoblastic leukaemia?. Lancet.

[63] Kozloski G.D., de Vito J.M., Kisicki J.C., Johnson J.B. (1992). The effect of food on the absorption of methotrexate sodium tablets in healthy volunteers. Arthritis Rheum..

[64] Baxter K., Preston C.L. (2012). Stockley’s Drug Interactions.

[65] McCracken G.H., Ginsburg C.M., Clahsen J.C., Thomas M.L. (1978). Pharmacologic evaluation of orally administered antibiotics in infants and children: Effect of feeding on bioavailability. Pediatrics.

[66] Okuno A., Taguchi T., Inyaku F., Yano K., Suzuki Y. (1983). Pharmacokinetics of propylthiouracil in children and adolescents with Graves disease after a single oral dose. Pediatr. Pharmacol. (N. Y.).

[67] Melander A., Wåhlin E., Danielson K., Hanson A. (1977). Bioavailability of propylthiouracil: Interindividual variation and influence of food intake. Acta Med. Scand..

[68] Pedersen S. (1986). Effects of food on the absorption of theophylline in children. J. Allergy Clin. Immunol..

[69] Jonkman J.H. (1989). Food interactions with sustained-release theophylline preparations. A review. Clin. Pharmacokinet..

[70] Lancaster D.L., Patel N., Lennard L., Lilleyman J.S. (2001). 6-Thioguanine in children with acute lymphoblastic leukaemia: influence of food on parent drug pharmacokinetics and 6-thioguanine nucleotide concentrations. Br. J. Clin. Pharmacol..

[71] Zent C., Smith P. (1995). Study of the effect of concomitant food on the bioavailability of rifampicin, isoniazid and pyrazinamide. Tuber. Lung Dis..

[72] Lin J.H. (1999). Role of pharmacokinetics in the discovery and development of indinavir. Adv. Drug Deliv. Rev..

[73] Neuvonen P.J. (1991). The effect of magnesium hydroxide on the oral absorption of ibuprofen, ketoprofen and diclofenac. Br. J. Clin. Pharmacol..

[74] Mattick L.R., Moyer J.C. (1983). Composition of apple juice. J. Assoc. Off. Anal. Chem..

[75] Allen L.V., Stiles M.L., Prince S.J., McLaury H.J., Sylvestri M.F. (1995). Stability of ramipril in water, apple juice, and applesauce. Am. J. Health-Syst. Pharm..

[76] Pao L.H., Zhou S.Y., Cook C., Kararli T., Kirchhoff C., Truelove J., Karim A., Fleisher D. (1998). Reduced systemic availability of an antiarrhythmic drug, bidisomide, with meal co-administration: relationship with region-dependent intestinal absorption. Pharm. Res..

[77] Reppas C., Eleftheriou G., Macheras P., Symillides M., Dressman J.B. (1998). Effect of elevated viscosity in the upper gastrointestinal tract on drug absorption in dogs. Eur. J. Pharm. Sci..

[78] Mimoz O., Binter V., Jacolot A., Edouard A., Tod M., Petitjean O., Samii K. (1998). Pharmacokinetics and absolute bioavailability of ciprofloxacin administered through a nasogastric tube with continuous enteral feeding to critically ill patients. Intensive Care Med..

[79] Wallace A.W., Amsden G.W. (2002). Is it really OK to take this with food? Old interactions with a new twist. J. Clin. Pharmacol..

[80] Eshra A.G., Etman M.A., Naggar V.F. (1988). Effect of milk and food on the bioavailability of ketoprofen in man. Int. J. Pharm..

[81] De Lemos M.L., Hamata L., Jennings S., Leduc T. (2007). Interaction between mercaptopurine and milk. J. Oncol. Pharm. Pract..

[82] Lourenço R. (2001). Enteral feeding: Drug/nutrient interaction. Clin. Nutr..

[83] Agency E.M. (2008). ICH Topic Q4B Annex 5 Disintegration Test General Chapter. EMEA/CHMP/ICH/308895/2008.

[84] Dalenback J., Abrahamson H., Bjornson E., Fandriks L., Mattsson A., Olbe L., Svennerholm A., Sjovall H. (1998). Human duodenogastric reflux, retroperistalsis, and MMC. Am. J. Physiol..

[85] Sanaka M., Kuyama Y., Shimomura Y., Qi J.F., Okamura S., Hao Y., Jainguo C., Mineshita S. (2002). Gastric emptying of liquids is delayed by co-ingesting solids: A study using salivary paracetamol concentrations. J. Gastroenterol..

[86] Abrahamsson B., Albery T., Eriksson A., Gustafsson I., Sjöberg M. (2004). Food effects on tablet disintegration. Eur. J. Pharm. Sci..

[87] Brouwers J., Anneveld B., Goudappel G.-J., Duchateau G., Annaert P., Augustijns P., Zeijdner E. (2011). Food-dependent disintegration of immediate release fosamprenavir tablets: *In vitro* evaluation using magnetic resonance imaging and a dynamic gastrointestinal system. Eur. J. Pharm. Biopharm..

[88] Smith S., Collaku A., Heaslip L., Yue Y., Starkey Y.-Y., Clarke G., Kronfeld N. (2012). A new rapidly absorbed paediatric paracetamol suspension. A six-way crossover pharmacokinetic study comparingthe rate and extent of paracetamol absorption from a new paracetamol suspension with two marketed paediatric formulations. Drug Dev. Ind. Pharm..

[89] Charkoftaki G., Kytariolos J., Macheras P. (2010). Novel milk-based oral formulations: Proof of concept. Int. J. Pharm..

[90] Kytariolos J., Charkoftaki G., Smith J.R., Voyiatzis G., Chrissanthopoulos A., Yannopoulos S.N., Fatouros D.G., Macheras P. (2013). Stability and physicochemical characterization of novel milk-based oral formulations. Int. J. Pharm..

[91] Research, F. a. D.A. C. f. D.E. a. NDA 202067 Clobazam. http://www.accessdata.fda.gov/drugsatfda_docs/nda/2011/202067Orig1s000ClinPharmR.pdf.

[92] Chun A.H.C., Erdman K., Zhang Y., Achari R., Cavanaugh J.H. (2000). Effect on bioavailability of admixing the contents of lansoprazole capsules with selected soft foods. Clin. Ther..

[93] Chun A.H.C., Erdman K., Chiu Y.-L., Pilmer B.L., Achari R., Cavanaugh J.H. (2002). Bioavailability of lansoprazole granules administered in juice or soft food compared with the intact capsule formulation. Clin. Ther..

[94] Fay M.A., Sheth R.D., Gidal B.E. (2005). Oral absorption kinetics of levetiracetam: The effect of mixing with food or enteral nutrition formulas. Clin. Ther..

[95] Pentikis H.S., Simmons R.D., Benedict M.F., Hatch S.J. (2002). Methylphenidate Bioavailability in Adults When an Extended-Release Multiparticulate Formulation Is Administered Sprinkled on Food or as an Intact Capsule. J. Am. Acad.Child Adolesc. Psychiatry.

[96] Eliot L., Butler J., Devane J., Loewen G. (2002). Pharmacokinetic evaluation of a sprinkle-dose regimen of a once-daily, extended-release morphine formulation. Clin. Ther..

[97] Thyssen A., Solanki B., Treem W. (2012). Randomized, Open-Label, Single-Dose, Crossover, Relative Bioavailability Study in Healthy Adults, Comparing the Pharmacokinetics of Rabeprazole Granules Administered Using Soft Food or Infant Formula as Dosing Vehicle Versus Suspension. Clin. Ther..

[98] Foulds G., Luke D.R., Teng R., Willavize S.A., Friedman H., Curatolo W.J. (1996). The absence of an effect of food on the bioavailability of azithromycin administered as tablets, sachet or suspension. J. Antimicrob. Chemother..

[99] Kovarik J.M., Noe A., Berthier S., McMahon L., Langholff W.K., Marion A.S., Hoyer P.F., Ettenger R., Rordorf C. (2003). Clinical Development of an Everolimus Pediatric Formulation: Relative Bioavailability, Food Effect, and Steady-State Pharmacokinetics. J. Clin. Pharmacol..

[100] FDA Centre for Drug Evaluation and Research NDA 22–571 Glycopyrrolate. http://www.accessdata.fda.gov/drugsatfda_docs/nda/2010/022571Orig1s000ClinPharmR.pdf.

[101] FDA Centre for Drug Evaluation and Research NDA 20–944 Advil (ibuprofen). http://www.accessdata.fda.gov/drugsatfda_docs/nda/98/20944_Advil_biopharmr.pdf.

[102] FDA Centre for Drug Evaluation and Research Application 020778 Viracept oral powder. http://www.accessdata.fda.gov/drugsatfda_docs/nda/97/020778ap.pdf.

[103] FDA Centre for Drug Evaluation and Research NDA 21–818,21–498/S-003 Nitazoxanide. http://www.accessdata.fda.gov/drugsatfda_docs/nda/2005/021818s000_ClinPharmR.pdf.

[104] Ibarra M., Fagiolino P., Vázquez M., Ruiz S., Vega M., Bellocq B., Pérez M., González B., Goyret A. (2012). Impact of food administration on lopinavir-ritonavir bioequivalence studies. Eur. J. Pharm. Sci..

[105] FDA Centre for Drug Evaluation and Research NDA 201367 Rufinamide. http://www.accessdata.fda.gov/drugsatfda_docs/nda/2011/201367Orig1s000ClinPharmR.pdf.

[106] FDA Centre for Drug Evaluation and Research NDA 20–990 Sertraline HCl. http://www.accessdata.fda.gov/drugsatfda_docs/nda/99/20990_Zoloft_biopharmr.pdf.

[107] FDA Centre for Drug Evaluation and Research NDA 20844 (S031) Topiramate. http://www.fda.gov/downloads/Drugs/DevelopmentApprovalProcess/DevelopmentResources/ucm129621.pdf.

[108] FDA Centre for Drug Evaluation and Research NDA 21–822 Tipranavir. http://www.accessdata.fda.gov/drugsatfda_docs/nda/2008/021822s000ClinPharmr.pdf.

[109] Shah T., Tse A.P.Y., Gill H., Wong I.C.K., Sutcliffe A., Gringras P., Appleton R., Tuleu C. (2008). Administration of melatonin mixed with soft food and liquids for children with neurodevelopmental difficulties. Dev. Med. Child Neurol..

[110] Singh B.N. (2005). A quantitative approach to probe the dependence and correlation of food-effect with aqueous solubility, dose/solubility ratio, and partition coefficient (Log P) for orally active drugs administered as immediate-release formulations. Drug Dev. Res..

[111] Amidon G.L., Lennernas H., Shah V.P., Crison J.R. (1995). A theoretical basis for a biopharmaceutic drug classification: The correlation of *in vitro* drug product dissolution and *in vivo* bioavailability. Pharm. Res..

[112] Galia E., Nicolaides E., Hörter D., Löbenberg R., Reppas C., Dressman J.B. (1998). Evaluation of various dissolution media for predicting In vivo performance of class I and II drugs. Pharm. Res..

[113] Ashby L.J., Beezer A.E., Buckton G. (1989). *In vitro* dissolution testing of oral controlled release preparations in the presence of artificial foodstuffs. I. Exploration of alternative methodology: microcalorimetry. Int. J. Pharm..

[114] Sunesen V.H., Pedersen B.L., Kristensen H.G., Müllertz A. (2005). *In vivo in vitro* correlations for a poorly soluble drug, danazol, using the flow-through dissolution method with biorelevant dissolution media. Eur. J. Pharm. Sci..

[115] Kataoka M., Masaoka Y., Sakuma S., Yamashita S. (2006). Effect of food intake on the oral absorption of poorly water-soluble drugs: *In vitro* assessment of drug dissolution and permeation assay system. J. Pharm. Sci..

[116] Blanquet S., Zeijdner E., Beyssac E., Meunier J.P., Denis S., Havenaar R., Alric M. (2004). A dynamic artificial gastrointestinal system for studying the behavior of orally administered drug dosage forms under various physiological conditions. Pharm. Res..

[117] Vardakou M., Mercuri A., Barker S.A., Craig D.Q., Faulks R.M., Wickham M.S. (2011). Achieving antral grinding forces in biorelevant *in vitro* models: Comparing the USP dissolution apparatus II and the dynamic gastric model with human *in vivo* data. AAPS PharmSciTech.

[118] Havenaar R., Anneveld B., Hanff L.M., de Wildt S.N., de Koning B.A.E., Mooij M.G., Lelieveld J.P.A., Minekus M. (2013). *In vitro* gastrointestinal model (TIM) with predictive power, even for infants and children?. Int. J. Pharm..

[119] Wagner C., Jantratid E., Kesisoglou F., Vertzoni M., Reppas C., Dressman J.B. (2012). Predicting the oral absorption of a poorly soluble, poorly permeable weak base using biorelevant dissolution and transfer model tests coupled with a physiologically based pharmacokinetic model. Eur. J. Pharm. Biopharm..

[120] Barrett J.S., Della Casa Alberighi O., Laer S., Meibohm B. (2012). Physiologically Based Pharmacokinetic (PBPK) Modeling in Children. Clin. Pharmacol. Ther..

